# Thrombus straddling the patent foramen ovale: a case report

**DOI:** 10.3389/fmed.2026.1840190

**Published:** 2026-07-07

**Authors:** Qin Jiangyan, Dan Li, Shi Mengrong, Hu Zitao

**Affiliations:** 1 Department of Radiology, Yan’an University Affiliated Hospital, Yanan, China; 2 Department of General Medicine, Yan’an University Affiliated Hospital, Yanan, China

**Keywords:** case report imaging finding, impending paradoxical embolus, patent foramen ovale, pulmonary embolism, thrombus, thrombus in transit

## Abstract

**Background:**

A transseptal thrombus straddling the patent foramen ovale (TSFO) is a rare but potentially life-threatening imaging finding that often serves as direct evidence of paradoxical embolism. Given its dynamic and transient nature, timely recognition is crucial to prevent systemic embolism.

**Case summary:**

We report an 81-year-old woman admitted for acute dyspnea. Transthoracic echocardiography (TTE) on admission revealed no intracardiac thrombus. However, contrast-enhanced cardiac computed tomography (CT) performed the following day unexpectedly demonstrated a linear hypodense lesion spanning the interatrial septum at the fossa ovalis, exhibiting the “straddling sign” and confirming the diagnosis of TSFO. Following shared decision-making with the patient, anticoagulation alone was deemed the optimal management. Telephone follow-ups at 1 and 3 months, and up to 2 years post-discharge, demonstrated a favorable clinical recovery.

**Discussion:**

This case highlights the diagnostic value of contrast-enhanced CT angiography in identifying TSFO in the setting of acute pulmonary embolism. Although TSFO is rare, it represents a “window period” manifestation of paradoxical embolism, necessitating heightened vigilance from both radiologists and clinicians. Early identification and initiation of anticoagulant therapy can effectively prevent systemic thromboembolic events and improve prognosis. Due to its dynamic and transient nature, imaging capture particularly on CT angiography is exceedingly rare. This case demonstrates a clear image of a transseptal thrombus, holding significant clinical and educational value.

## Introduction

TSFO is a rarely visualized imaging finding. The foramen ovale is a physiological shunt present during fetal development that allows right-to-left atrial blood flow. At birth, rising left atrial pressure leads to functional closure of the foramen ovale, with anatomical closure typically occurring within the first year of life. When the foramen remains open beyond the age of 3 years, it is termed a patent foramen ovale (PFO). In the presence of a PFO, venous emboli may cross from the right to the left atrium. If the thrombus is relatively large and the PFO diameter is small, the clot may become impacted within the foramen during transit. Imaging evidence of a transseptal thrombus provides compelling support for the paradoxical embolism theory in PFO-associated stroke. We report a case of TSFO to aid clinicians in the diagnosis and management of this critical condition.

## Case presentation

An 81-year-old woman had experienced recurrent episodes of exertional chest tightness and dyspnea over the preceding 6 months. Half a month prior to admission, her symptoms markedly worsened following an upper respiratory tract infection. She received symptomatic treatment including antibiotics and diuretics at a community hospital, with partial improvement. Over the 4 days before presentation, she developed worsening dyspnea and chest tightness without apparent trigger, now limiting her ability to lie flat at night.

Doppler ultrasound of the lower extremity veins performed at a community hospital on October 24, 2023, thrombosis was observed in the bilateral anterior tibial, posterior tibial, and peroneal veins, and the left femoral vein. She was subsequently referred to our institution on October 25, 2023.

On admission, the patient was in moderate respiratory distress, maintaining a semi-recumbent position. She was unable to lie flat. Her heart rate was 90 beats per minute with regular rhythm, blood pressure was 180/110 mmHg, and the aortic component of the second heart sound (A2) was louder than the pulmonic component (P2). Laboratory findings included: white blood cell count, 11.15 × 10^9^/L; C-reactive protein, 28.09 mg/L; high-sensitivity C-reactive protein, > 10.0 mg/L; fibrinogen degradation products, 16.99 μg/mL; high-sensitivity troponin I, 45.9 pg/mL; D-dimer, 7.99 μg/mL; and B-type natriuretic peptide, 2850 pg/mL. Arterial blood gas analysis (on room air) showed: pH 7.417, PaO_2_ 61.5 mmHg, PaCO_2_ 36.3 mmHg, SaO_2_ 89.6%, and PaO_2_/FiO_2_ ratio 212 mmHg.

**FIGURE 1:**
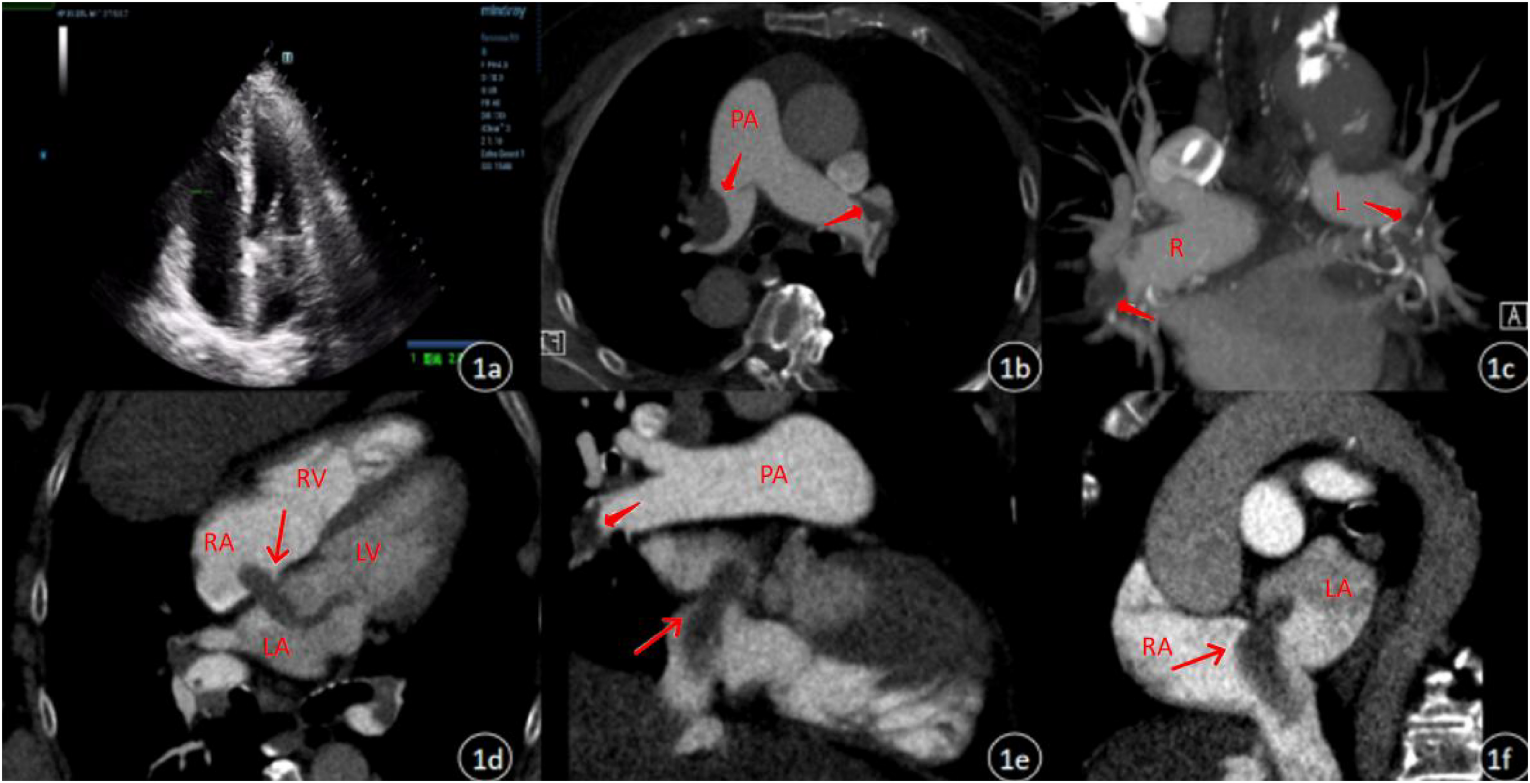
**(a)** Cardiac ultrasound demonstrates normal intracardiac structures and chamber dimensions; **(b,c)** CTA reveals thrombus in the left and right pulmonary arteries; **(d–f)** CTA shows an embedded, thin, earthworm-like thrombus visible in the foramen ovale region. RA, right atrium; LA, left atrium; RV, right ventricle; LV, left ventricle; PA, pulmonary artery.

Transthoracic echocardiography performed on October 26, 2023, showed normal cardiac chamber dimensions and great vessel diameters (Figure 1a). Left ventricular ejection fraction was 59% (fractional shortening 32%). Echocardiography revealed no regurgitation in the mitral, tricuspid, or pulmonary valves, and no intracardiac masses were observed. Color Doppler ultrasonography of the lower extremities revealed thrombosis in the bilateral anterior tibial, posterior tibial, and peroneal veins, as well as in the left femoral vein. On October 27, 2023, pulmonary artery computed tomography angiography (CTA) revealed: (1) multiple bilateral pulmonary emboli (Figures 1b, c); and (2) a patent foramen ovale with a straddling thrombus (Figures 1d–f). The thrombus appeared as a thin, earthworm-like hypodense structure embedded within the fossa ovalis, with its central portion traversing the septum and the ends free-floating in both atria, without attachment to the atrial walls.

Notably, TTE performed the day before had shown no abnormal echoes in the interatrial septum. This temporal discrepancy suggests that the thrombus became lodged in the PFO between the two imaging studies, supporting the diagnosis of an acute TSFO. Following multidisciplinary deliberation and shared decision-making with the patient, anticoagulation alone was deemed the optimal strategy after weighing the high risks of thrombolysis and surgery against the potential benefits of anticoagulation. Consequently, rivaroxaban (15 mg twice daily) was initiated. After 1 week of anticoagulation, her symptoms improved significantly. She was discharged in stable condition with instructions to continue rivaroxaban 15 mg twice daily for 2 weeks, then transition to 20 mg once daily. Outpatient follow-up was scheduled at 1, 3, 6, and 12 months. Telephone assessments at 1 and 3 months post-discharge confirmed sustained clinical stability without recurrence of dyspnea, chest discomfort, or other complications. Telephone follow-ups at 6 months, 1 year, and 2 years were conducted, during which the patient’s family reported strict adherence to the prescribed oral anticoagulation regimen. No major bleeding events occurred. Furthermore, the patient remained free of thromboembolism-related symptoms, such as chest tightness, chest pain, dyspnea, syncope, or limb numbness and pain, and maintained a stable daily functional status.

## Discussion

TSFO is an exceptionally rare imaging finding, with most current reports being case series (1–5). The foramen ovale normally closes functionally shortly after birth due to increased left atrial pressure from pulmonary venous return. Anatomical closure occurs in approximately 75% of individuals; the remaining 25% retain a probe-patent foramen ovale into adulthood (6). When right atrial pressure transiently exceeds left atrial pressure-as during coughing, Valsalva maneuver, or acute right heart strain from pulmonary embolism-a right-to-left shunt can occur, enabling venous thrombi to enter the systemic circulation (7).

In this case, acute pulmonary embolism likely elevated right heart pressures, triggering transient right-to-left shunting through a pre-existing PFO and resulting in thrombus impaction. The absence of the thrombus on TTE one day prior to CTA underscores the dynamic nature of this phenomenon.

Transesophageal echocardiography (TEE) remains the primary diagnostic modality for TSFO (8). Regrettably, due to the advanced age, frailty, and inability to withstand the supine position, the patient did not undergo TEE examination, thereby imposing constraints on this case. CTA offers the unique advantage of simultaneously evaluating pulmonary vasculatureand the interatrial septum. The “straddling sign” on CTA characterized by a thrombus bridging the atria with minimal wall adherence is highly specific for TSFO (9).

The presence of a transseptal thrombus strongly supports the paradoxical embolism mechanism in cryptogenic stroke (CS). Indeed, paradoxical embolism accounts for 2–16% of all arterial embolic events and is the leading cause of CS in patients with PFO Seo et al. reported that among 194 patients with PFO associated saddle thrombi, 37.6% had suffered prior systemic embolism, most commonly cerebral infarction, about 24.7% (10, 11). Fortunately, our patient exhibited no evidence of systemic embolization beyond the pulmonary circulation.

Currently, no randomized controlled trials exist to guide the clinical management of TSFO. Li et al. (12) recently explored individualized treatment strategies for TSFO based on imaging characteristics and clinical history. Current clinical practice generally favors the immediate initiation of anticoagulation to prevent thrombus embolization and paradoxical embolism. For hemodynamically unstable patients, thrombolysis or surgical embolectomy may be considered. In our case, although the patient presented with dyspnea on admission, the blood pressure was 180/110 mmHg, heart rate was 90 bpm with a regular rhythm, and TTE revealed a LVEF of 59% (fractional shortening, 32%). Combined with a mildly elevated cTnI, these findings indicated hemodynamic stability. Although CTPA revealed multiple emboli, there was no evidence of a “saddle embolus” or complete occlusion of the main pulmonary artery. Furthermore, due to advanced age and other factors, the patient was at high risk for bleeding, which would have made thrombolytic therapy highly susceptible to hemorrhagic transformation.

Following multidisciplinary deliberation and shared decision-making with the patient, anticoagulation monotherapy was deemed the optimal strategy after weighing the high risks of thrombolysis and surgery against the potential benefits of anticoagulation. High-intensity anticoagulation with rivaroxaban was promptly initiated to stabilize the thrombus, preventing further embolization or propagation while avoiding thrombolysis-associated bleeding complications. The patient’s symptoms improved significantly after 1 week of treatment. Although the patient explicitly declined further in-person hospital visits post-discharge, precluding the acquisition of subsequent clinical and imaging data, we conducted telephone follow-ups for up to 2 years. During this period, the patient remained clinically stable and free of thromboembolism-related symptoms, such as chest tightness, chest pain, dyspnea, syncope, or extremity numbness and pain. This report provides real-time imaging evidence of acute thrombus impaction in a PFO secondary to pulmonary embolism, offering direct validation of the paradoxical embolism pathway. It also emphasizes the critical role of multimodal imaging, particularly CTA, in capturing this fleeting yet life-threatening condition.

## Data Availability

The raw data supporting the conclusions of this article will be made available by the authors, without undue reservation.
